# Postoperative Hypoparathyroidism: Prognosis, Prevention, and Treatment (Review)

**DOI:** 10.17691/stm2020.12.2.13

**Published:** 2020

**Authors:** A.A. Melikyan, A.V. Menkov

**Affiliations:** Surgeon, Nizhny Novgorod Regional Clinical Hospital named after N.A. Semashko, 190 Rodionova St., Nizhny Novgorod, 603126, Russia; Professor, Department of General, Operative Surgery and Topographic Anatomy named after A.I. Kozhevnikov, Privolzhsky Research Medical University, 10/1 Minin and Pozharsky Square, Nizhny Novgorod, 603005, Russia

**Keywords:** hypoparathyroidism, hypocalcemia, thyroid gland, parathyroid hormone, parathyroid glands, thyroidectomy

## Abstract

This review summarizes the results of studies concerning the problem of post-surgical hypoparathyroidism, a common complication of thyroid gland surgery, decreasing the quality of life in patients and, in some cases, leading to disability.

A search for publications was carried out in electronic databases Web of Science, Scopus, Academic Search Complete (EBSCO), eLIBRARY, using keywords. The search depth was 7–10 years. Prevalence of post-surgical hypoparathyroidism was evaluated, the pathogenetic causes of the disease development, its clinical forms, methods of diagnosis and treatment were studied. It has been found that there is no single algorithm for analyzing the prognostic factors for the development of this pathological condition. It is emphasized that drug therapy of post-surgical hypoparathyroidism has a number of adverse effects. Therefore, the issues of prevention and surgical correction are of particular relevance. However, controversial opinions of contemporary authors about their clinical effectiveness determine the scientific and practical significance of further research on these issues.

## Introduction

Surgical treatment of thyroid gland (TG) diseases is one of the priority areas in modern medical science as the number of patients with thyroid pathology is constantly increasing. Therefore, the prevalence of thyroidopathies in the Russian Federation averages at 10%, and morbidity rate is 50 new cases per 100 000 population per year [1–3]. Consequently, the number of surgical interventions on the thyroid gland inevitably increases. Owing to the possibility of conducting adequate replacement therapy with synthetic analogues of levothyroxine, thyroidectomy has become the operation of choice in treatment of not only thyroid malignancies, but also most benign thyroid diseases [2, 3]. However, this type of surgical intervention can lead to failure of the parathyroid glands (PTG) function, caused by their injury or impaired blood supply [4–6]. This pathological condition is referred to as “post-surgical hypoparathyroidism” (PSHP) and characterized by decreased levels of parathyroid hormone (PTH) in the blood, the development of hypocalcemia and hyperphosphatemia.

Asari et al. [7] understand PSHP as a documented decrease in serum calcium levels after surgery below 1.9 mmol/L, regardless of the presence of hypocalcemia symptoms, or as a case when a patient has neuromuscular manifestations, and postoperative serum calcium levels range from 1.9 to 2.1 mmol/L.

In PSHP, it is often impossible to compensate for calcium status impairment with drugs adequately [8, 9]. Clinical manifestations of PSHP, such as multiple calcinosis, cataracts, tetany, gastrointestinal diseases, lead to decreased quality of life in patients, and in some cases to disability [9**–**12].

## Epidemiology, pathogenesis, and clinical picture

The prevalence of PSHP is 20 cases per 100,000 population, according to authors from Denmark [4]. American colleagues report in their study that this figure amounts to 32 cases per 100,000 people [6]. The incidence of PSHP depends on the extent of surgery on the thyroid gland and the experience of the surgeon. In specialized centers, hypocalcemia is observed after thyroidectomy in 1–7.5% of patients [11, 13]. At the same time, different authors report the incidence of PSHP to range from 5 to 17% [4–7]. Most often, hypocalcemia develops after operations performed for diffuse toxic goiter (Graves–Basedow disease) and thyroid cancer [5, 14]. This is probably associated with metabolic disorders against the background of accelerated bone tissue remodeling in diffuse toxic goiter [15, 16], the technical aspects of surgical intervention and the need for lymphadenectomy in patients with thyroid tumors [17, 18]. As a rule, a decrease in calcium levels is recorded 24–48 h after surgery [9, 16, 19]. However, the manifestations of PSHP can be observed in patients several months and even years after surgery [16, 19, 20]. Kamath and Rao [20] report a case of clinical manifestation of PSHP 15 years after thyroid resection. The pathogenic causes of late PSHP are understood incompletely and seem to be associated with progressive atrophy of PTG due to their insufficient blood supply [16].

Hypocalcemia can be temporary and stop within two months after the operation (transient PSHP). The incidence of transient PSHP ranges from 18 to 39% [6, 15, 21, 22]. Temporary hypocalcemia most often develops in patients operated on for benign thyroid diseases and is considered a consequence of post-traumatic (ischemic) thyroid dysfunction [6, 7, 15, 22].

The sign of *permanent PSHP* is hypocalcemia in combination with low levels of PTH during 6 months or more after surgery [16, 19, 22, 23]. This type of PSHP is observed in 1–7% of patients who underwent thyroidectomy in combination with paratracheal lymphadenectomy or repeated interventions on the thyroid gland [17, 18, 22–24]. Inadequate width of surgical margins and surgical injury can lead not only to PTG devascularization, but also to their accidental removal [22, 23, 25]. According to Lorente-Poch et al. [26], the risk of permanent PSHP is closely related to the number of PTG remaining after surgery: 16%, if one or two glands function; 6%, if there are three; and 2.5%, if four glands are preserved.

Along with the extent of surgery and nature of the pathology, some authors refer to age and gender of patients as predisposing factors for the development of PSHP [7, 22]. However, Erbil et al. [27] found no significant correlation between age and PSHP incidence rates.

Many researchers consider vitamin D deficiency present in patients before surgery to be the most significant risk factor for PSHP [27–31]. For example, according to Simakina et al. [32], preoperative vitamin 25(OH)D3 levels <15 ng/mL are a reliable predictor of PSHP. Therefore, it seems appropriate to introduce preoperative screening of this parameter, especially in patients with thyroid tumors and diffuse toxic goiter.

The clinical picture of PSHP is determined by the severity of hypocalcemia and hyperphosphatemia [9, 33]. With serum calcium level decreasing to 2 mmol/L, PSHP often develops without any clinical manifestations (*subclinical PSHP*) [9, 33]. Progressing calcium status impairment results in increased neuromuscular excitability and general vegetative reactivity [9, 34–36], which manifest as paresthesia and numbness of the distal extremities and face, twitching of the facial muscles (Chvostek sign), flexion of the wrist joint, metacarpophalangeal joints, hyperextension of the interphalangeal joints (main d’accoucheur). In some cases, bronchial and laryngospasm are observed [9, 34, 36, 37]. Permanent PSHP is characterized by mental changes (memory impairment, sleep disturbance, depressive states). Calcium-phosphorus metabolism disturbance may lead to cataracts, teeth enamel defects, dry skin, nail brittleness, and impaired hair growth [9, 34, 37, 38]. Increased phosphorus excretion and reduced calcium reabsorption in the proximal nephron leads to the formation of kidney stones and subsequent renal failure [9, 33, 37, 39]. Hypocalcemic myocardiopathy is accompanied by expansion of the heart chambers and reduced ejection fraction: prolongation of the QT interval, tachycardia, and ventricular fibrillation appear [39–41].

## Diagnosis and prognosis of post-surgical hypoparathyroidism

Diagnosis of PSHP is based on the measurement of calcium (albumin-adjusted total calcium and ionized calcium), phosphate and PTH levels in serum [35, 42–45]. Asari et al. [7] believe that one of the criteria for PSHP development is decreased PTH levels in the first day after surgery — <15 pg/ml. Ritter et al. [22] point to PTH values <10 pg/ml. In their opinion, PSHP should be considered as permanent if PTH value does not recover within 1 year after surgery or its level is greater than or equal to 10 pg/ml, but the patient still needs medical correction of hypocalcemia symptoms.

It was recommended at the European Congress of Endocrinology (May 2015) to determine the levels of albumin, magnesium, and vitamin D3 in serum [46, 47]. Hypomagnesemia may cause a decrease in PTH secretion, while vitamin D deficiency is a risk factor for the development of PSHP [27–29, 48].

Predicting the development of PSHP and adequate conservative therapy can either prevent the occurrence of this complication or minimize the severity of the clinical manifestations of hypocalcemia [42–44, 49–52].

Lazard et al. [45] have developed an algorithm for early detection of hypocalcemia based on the dynamics of serum calcium levels measured before surgery and in the early hours (6–48 h) of the postoperative period. According to Wang and colleagues [53], PTH levels allow predicting the development of PSHP more accurately than calcium levels. Chindavijak [54] found a statistically significant decrease in PTH levels 20 min after thyroidectomy in patients who developed PSHP soon afterwards as compared to patients without calcium status impairment after surgery. Sensitivity and specificity of the proposed method were 85 and 80%, respectively. Similar results were obtained by Proczko-Markuszewska et al. [55] and Russian researchers [56]. However, Promberger et al. [43] found that even the normal values of the postoperative PTH level may be insufficient to maintain adequate calcium metabolism, therefore a combined analysis of several PSHP predictors is required.

Sam et al. [57] show in their pilot study that vitamin D deficiency can lead to increased concentrations and a slower decrease in PTH levels after thyroidectomy. The authors emphasize the need to develop correlation scales for the relationship between the studied parameters and the calculated differences in PTH values before and after surgery.

Other authors [49, 50] found no dependence of postoperative calcium or PTH levels on the preoperative vitamin 25(OH)D3 level.

Thus, controversial opinions about the prognostic effectiveness of various factors causing PSHP and the lack of a single algorithm for their combined analysis require further investigation of this issue.

## Principles of drug therapy

Treatment of PSHP mainly aims at reducing hypocalcemia symptoms (target values of ionized calcium — 1.10–1.25 mmol/L, albumin-adjusted total calcium — 2.1–2.3 mmol/L), maintaining serum phosphate levels at the upper limit of the reference range, preventing hypercalciuria and the resulting development of nephrolithiasis, nephrocalcinosis and chronic renal failure [46, 47, 58–60].

To relieve the clinical manifestations of acute hypocalcemia, it is necessary to administer intravenous calcium salts (preferably calcium gluconate) simultaneously with active vitamin D metabolites. There should be daily monitoring of calcium status until the lower values of the serum calcium reference range are reached [9, 58, 59, 61, 62].

The standard therapy for permanent PSHP is oral calcium medications (carbonate or citrate, 1 to 3 g per day), metabolites (alfacalcidol — 0.5–3 μg/day or calcitriol — 0.25–2 μg/day) and native vitamin D (colecalciferol — up to 2000 IU/day) [58, 59, 61, 63–65]. To prevent hyperphosphatemia, diet is prescribed: the limited use of phosphorus-containing food (dairy products, nuts, beer) and carbonated drinks impairing calcium absorption in the intestine [9, 63, 64]. Unfortunately, long-term treatment with calcium and vitamin D medications can lead to hypercalciuria (even in normocalcemia) with the resulting nephrolithiasis, nephrocalcinosis and renal failure, as well as systemic calciphylaxis such as intracranial calcification [63, 65–67]. Besides, patients have resistance to vitamin D or malabsorption syndrome [59]. Therefore, PTH analogues — teriparatide (1-34 N-fragment of the PTH molecule) and Natpara (recombinant human PTH 1-84) — have been recently used in the combined therapy for severe PSHP [68–71].

Hormonal drugs can reduce the daily dose of calcium by 50% and achieve normocalciuria [68–71]. However, it is not always possible to restore the calcium and phosphorus balance in the body completely, there is a risk of developing osteosarcomas [69, 70, 72]. The European working group on the management of patients with chronic hypoparathyroidism recommends against the routine use of recombinant PTH, unless their use is indicated individually in severe cases refractory to traditional therapy [46, 47].

Thus, drug therapy for PSHP, including the use of PTH analogues, has certain drawbacks. In this regard, the issues of prevention and surgical correction of PSHP are relevant.

## Prevention and surgical correction methods

The cornerstone of preventive measures is improvement of intraoperative visualization and mobilization of PTG during surgery.

The routine method for localization of PTG during thyroidectomy is staining the glands through intravenous administration of methylene blue [73]. However, this method has not become a common practice due to the lack of specificity [74]. Currently, 5-aminolevulinic acid (5-ALA) is preferred. Accumulating this drug, PTG acquire a pink-orange color under exposure to polarized light [75–77]. Kirpa et al. [77] showed in their study that the use of this technique allows preventing the development of PSHP after thyroidectomy with lymphatic dissection in 91% of patients. To reduce the systemic effect of 5-ALA, Elbassiouny et al. [78] carried out an experiment to test the possibility of introducing nanovesicles containing this drug. McWade et al. [79] performed intraoperative detection of PTG using near-infrared laser fluorescence spectroscopy, which made it possible to detect PTG in 100% of patients. An animal experiment established the possibility of using indocyanine green, a specific dye, for infrared fluorescence visualization of PTG [80].

The first experience of making injections of carbon nanoparticles into the thyroid tissue seems interesting. In the process, thyroid tissue takes black color, while PTG do not change their color, which greatly facilitates their isolation during surgery [81, 82]. The use of precision optics provides invaluable assistance in mobilizing PTG and contributes to their minimal injury, thereby reducing PSHP incidence [83, 84].

To facilitate localization of PTG and reduce the degree of their devascularization, Duboshina et al. [85] recommend extracapsular injections of 2 to 4 ml actovegin and gliatilin on the side of each lobe. However, this method is mainly aimed at preventing damage to the recurrent laryngeal nerve and contains no technical solutions to allow PTG mobilization without disturbing their blood supply.

It was found in a number of studies [86, 87] that clear visualization of tissues during minimally invasive video-assisted interventions on TG helps to avoid PTG injury and thereby reduce the probability of developing PSHP. However, other authors point to the absence of significant difference in the incidence of PSHP after conventional and minimally invasive thyroidectomy [88–90]. Thus, the techniques for intraoperative visualization and mobilization of PTG need further improvement.

Transplantation technologies are considered the most promising methods for correcting PSHP, especially, if the disease is severe and persistent [91, 92]. The first experimental transplantation of TG and PTG tissue into the preperitoneal cellular tissue of a cat was performed in 1892 by Anton von Aizelsberg, demonstrating the absence of cramps in the thyroidectomized animal [93]. Currently, allotransplantation and autotransplantation have become widespread (urgent autotransplantation of PTG fragments performed during surgery and delayed autotransplantation of cryopreserved PTG) [91, 92]. Famà et al. [94] achieved a significant decrease in PSHP incidence having performed an emergency autotransplantation of PTG removed accidentally during thyroidectomy. They placed fragmented PTG tissue in a nutrient solution for 5 min, and then implanted it into the thickness of the sternocleidomastoid muscle. A positive effect of autologous PTG transplantation was achieved by surgeons from Kiev, who carried out administration of a suspension based on collagenase of minced PTG tissue into the thickness of the sternocleidomastoid muscle [95]. Evidence suggests that it is necessary to resort to autotransplantation of PTG when there is the slightest doubt about their viability. Ahmed and colleagues [96] believe that this approach allows retaining the function of at least one transplanted PTG, thereby avoiding permanent PSHP. Krausz et al. [97] point out that the success rate of autologous transplantation of cryopreserved PTG depends on transplant storage time and conditions. They argue that cryopreserved PTG should be stored for no more than two years, emphasizing that transplantation should be carried out in specialized centers.

Issues concerning the body area most suitable for transplantation are also discussed. In a comparative experiment on rats, Erikoglu et al. [98] found no significant differences as to the viability of PTG tissue during transplantation into various anatomical structures (the sternocleidomastoid muscle, the liver, peritoneum). In this context, the work of Popov et al. [99] is relevant. In the experiment on outbred dogs, the authors transplanted the removed PTG fragments into the lumen of a branch of the great saphenous vein, fixing the graft to the intima of the vessel. One month after the surgery, the values of calcium status and PTH levels of the animals appeared to be satisfactory and the viability of the transplanted tissue was morphologically confirmed. However, Tartaglia et al. [100] found that autologous PTG transplantation does not affect the incidence of postoperative hypocalcemia. Lorente-Poch et al. [101] came to a similar conclusion. A review published in 2018 shows that in some cases, autologous PTG transplantation can even have a negative effect on the course of PSHP [102].

Allotransplantation of cryopreserved PTG is most often used in cases of severe permanent PSHP when the consequences of immunosuppressive graft protection pose a lower risk for the patient than calcium replacement therapy. For example, Liu et al. [103] report a good clinical result of repeated reimplantations of cryopreserved PTG in a patient with severe long-term PSHP. To prevent the negative impact of immunosuppression, allotransplantation of macroencapsulated PTG cells has been proposed. Khryshchanovich and Ghoussein [104] have established the functional activity of a macrocapsule with donor PTG cells, made using polyvinylidene difluoride and implanted into the deep femoral artery.

There is considerable research interest in the experiments on cultivating the parathyroid cells on a three-dimensional collagen matrix, which allows preserving both the architecture of PTG tissue and its functional activity *in vitro* [105]. However, no reports on the clinical application of this technique have been found in the available literature.

## Conclusion

Post-surgical hypoparathyroidism is a common complication of surgery performed on the thyroid gland. Nevertheless, today there is no single algorithm for analyzing the prognostic factors for the development of this pathology. Drug therapy has a number of side effects. Therefore, prevention and surgical correction of post-surgical hypoparathyroidism are of particular relevance. However, controversial opinions of modern authors about their clinical effectiveness determine the scientific and practical significance of further investigation of these issues.
